# Automated Analysis of Flow Cytometry Data to Reduce Inter-Lab Variation in the Detection of Major Histocompatibility Complex Multimer-Binding T Cells

**DOI:** 10.3389/fimmu.2017.00858

**Published:** 2017-07-26

**Authors:** Natasja Wulff Pedersen, P. Anoop Chandran, Yu Qian, Jonathan Rebhahn, Nadia Viborg Petersen, Mathilde Dalsgaard Hoff, Scott White, Alexandra J. Lee, Rick Stanton, Charlotte Halgreen, Kivin Jakobsen, Tim Mosmann, Cécile Gouttefangeas, Cliburn Chan, Richard H. Scheuermann, Sine Reker Hadrup

**Affiliations:** ^1^Division of Immunology and Vaccinology, Veterinary Institute, Technical University of Denmark, Copenhagen, Denmark; ^2^Department of Immunology, Interfaculty Institute for Cell Biology, University of Tuebingen, Tuebingen, Germany; ^3^Department of Informatics, J. Craig Venter Institute, La Jolla, CA, United States; ^4^David H. Smith Center for Vaccine Biology and Immunology, University of Rochester Medical Center, Rochester, NY, United States; ^5^Department of Biostatistics and Bioinformatics, Duke University Medical Center, Durham, NC, United States; ^6^Human Longevity Inc., San Diego, CA, United States; ^7^Immudex Aps, Copenhagen, Denmark; ^8^Department of Pathology, University of California, San Diego, La Jolla, CA, United States

**Keywords:** major histocompatibility complex multimers, antigen-specific T cells, automated gating, computational analysis, major histocompatibility complex dextramers, flow cytometry

## Abstract

Manual analysis of flow cytometry data and subjective gate-border decisions taken by individuals continue to be a source of variation in the assessment of antigen-specific T cells when comparing data across laboratories, and also over time in individual labs. Therefore, strategies to provide automated analysis of major histocompatibility complex (MHC) multimer-binding T cells represent an attractive solution to decrease subjectivity and technical variation. The challenge of using an automated analysis approach is that MHC multimer-binding T cell populations are often rare and therefore difficult to detect. We used a highly heterogeneous dataset from a recent MHC multimer proficiency panel to assess if MHC multimer-binding CD8^+^ T cells could be analyzed with computational solutions currently available, and if such analyses would reduce the technical variation across different laboratories. We used three different methods, FLOw Clustering without K (FLOCK), Scalable Weighted Iterative Flow-clustering Technique (SWIFT), and ReFlow to analyze flow cytometry data files from 28 laboratories. Each laboratory screened for antigen-responsive T cell populations with frequency ranging from 0.01 to 1.5% of lymphocytes within samples from two donors. Experience from this analysis shows that all three programs can be used for the identification of high to intermediate frequency of MHC multimer-binding T cell populations, with results very similar to that of manual gating. For the less frequent populations (<0.1% of live, single lymphocytes), SWIFT outperformed the other tools. As used in this study, none of the algorithms offered a completely automated pipeline for identification of MHC multimer populations, as varying degrees of human interventions were needed to complete the analysis. In this study, we demonstrate the feasibility of using automated analysis pipelines for assessing and identifying even rare populations of antigen-responsive T cells and discuss the main properties, differences, and advantages of the different methods tested.

## Introduction

Antigen-specific T cell recognition is an essential component of the adaptive immune response fighting infectious diseases and cancer. The T cell receptor (TCR)-based recognition profile of a given T cell population can be determined through interaction with fluorescently labeled multimerized peptide major histocompatibility complexes (pMHC multimers) (1), enabling visualization of specific pMHC-responsive T cells by flow cytometry (2). This analysis has become state of the art for antigen-specific CD8^+^ T cell detection and is important for pathophysiological understanding, target discovery, and diagnosis of immune-mediated diseases. Detection of pMHC-responsive T cells is challenged by the low-avidity interaction between the TCR and the pMHC, often resulting in poor separation of fluorescent signals distinguishing the MHC multimer-binding from non-binding T cells (3). Additionally, a given antigen-specific T cell population is in most cases present at low frequencies in the total lymphocyte pool (4).

Substantial effort has been applied to optimize and standardize protocols for pMHC multimer staining of antigen-specific T cells to ensure the best possible signal-to-noise ratio in such T cell assays. The Immunoguiding Program of the European Association of Cancer Immunotherapy (CIP) has been actively involved in this process, and through a series of proficiency panels, identified the parameters largely impacting the variation in such assays (5–8). Among these, individual gating strategies lead to significant variation in final results determining the frequency of pMHC-responsive T cells (9). To minimize gating-associated variation and manual handling as well as to improve standardization, several automated analysis strategies have been developed to analyze flow cytometry data based on computational assessments of the different parameters involved (10, 11). These algorithms are based on computational identification of cell clusters in multidimensional space, taking into account all the different parameters applied to a certain cell type. Hence, they consider all associated parameters simultaneously, which forms an additional advantage compared with sequential 2D determinations of “positive” or “negative” categories, and consequently leads to a potentially improved identification of a given cell population.

The performance of automated analysis tools has been investigated in a number of challenges reported by the FlowCAP consortium (11–13), but such algorithms have so far not been evaluated for identification of MHC multimer-binding T cells. The aim of the present study was to test the feasibility and to report the experience of using automated analysis tools for identification of antigen-specific T cells. Tools were selected based on (a) the requirement of a user-friendly interface, making them accessible to flow cytometry users without computational expertise and (b) the described ability to detect rare cell populations. Three software solutions were chosen based on these criteria: FLOw Clustering without K (FLOCK) (14), Scalable Weighted Iterative Flow-clustering Technique (SWIFT) (15–17), and ReFlow (18, 19), but several others may be available having similar characteristics. FLOCK is a grid-based density clustering method for automated identification of cell populations from high-dimensional flow cytometry data, which is publicly accessible through the Immunology Database and Analysis Portal (ImmPort) at http://immport.niaid.nih.gov (now moved to https://www.immportgalaxy.org/). SWIFT is a model-based clustering method that is specifically developed to identify rare cell populations. The algorithm goes through three stages of fitting the cell populations to Gaussian distributions, splitting, and merging the populations to reach unimodality. The clustered output files given by SWIFT can either be analyzed by manual cluster gating or by automatically analyzing the cluster output. It is publicly available through http://www.ece.rochester.edu/projects/siplab/Software/SWIFT.html but requires Matlab software. ReFlow is a repository and automated analysis platform for flow cytometry data that is currently available as open source with web-based access and shared GPU computation (18, 19). It employs the hierarchical Dirichlet process Gaussian mixture model that naturally generates an aligned data model to capture both commonalities and variations across multiple samples, for the identification of unique cell subsets in an automated fashion (19). We evaluated the selected algorithms for their ability to identify pMHC multimer-binding T cells compared with manual gating, using data from a recent MHC multimer proficiency panel organized by Immudex^1^ in collaboration with CIP.^2^ We analyzed MHC Dextramer™ staining of T cells recognizing two different virus-derived epitopes [Epstein–Barr virus (EBV) HLA-A*0201/GLCTLVAML and influenza (FLU) HLA-A*0201/GILGFVFTL] in peripheral blood mononuclear cells (PBMCs) from two healthy donors. Furthermore, data from two sets of spike-in samples were used. The overall goal was to evaluate the feasibility and limit of detection of these three different algorithms that are readily available to flow users without pre-existing computational expertise.

## Materials and Methods

### Production of MHC Multimers

HLA-B*0702/TPRVTGGGAM monomers used in the spike-in 1 experiment were generated using UV-mediated peptide exchange as previously described (20). In short, HLA-B*0702 monomers carrying a UV-sensitive peptide were mixed with TPRVTGGGAM peptide in a final concentration of 100 µg/ml monomer and 200 µM peptide and kept under UV light for an hour. The resulting HLA-B*0702/TPRVTGGGAM monomers were then multimerized using phycoerythrin (PE)-streptavidin (BD Biosciences). The multimers were frozen at −80°C in freezing buffer giving a final multimer concentration of 10 µg/ml with 0.5% Bovine Serum Albumin (Sigma-Aldrich) and 5% glycerol (Fluka).

For the spike-in 2 experiment, HLA-A*0201/NLVPMVATV and HLA-A*0201/GILGFVFTL monomers were generated using classical refolding (1) and multimerized using streptavidin-PE or streptavidin-allophycocyanin (APC) (Life Technologies), respectively, at a 4:1 molar ratio. After the addition of 1 mM biotin (Sigma-Aldrich), the multimers were aliquoted and frozen at −80°C in a freezing solution containing 1.7% human serum albumin (Albiomin^®^, Biotest, Dreieich, Germany), 0.07% sodium azide, 3.4× protease inhibitor (Complete™, Sigma-Aldrich), 42% v/v glycerol (Roth), and 7 mMTBS, such that the final mixture contained 14% (v/v) glycerol (7). The stock concentrations of PE- and APC-conjugated multimers were 310 and 485 µg/ml, respectively.

### Donor Material

Peripheral blood mononuclear cells from healthy donors were obtained from buffy coats (blood products) collected at the local blood bank. All procedures were approved by the local Scientific Ethics Committee. PBMCs were isolated from buffy coats by density centrifugation on Lymphoprep (Axis-Shield PoC), and cryopreserved at −150°C in fetal calf serum (FCS; Gibco) + 10% DMSO.

### Spike-in Cell Samples

FCS files from two different spike-in experiments were used in this study, spike-in 1 and spike-in 2. For spike-in 1, one PBMC sample from donor BC260 (HLA-B*0702 positive) carrying a CD8 T cell response of 1.7% of single, live lymphocytes against the cytomegalovirus (CMV) HLA-B*0702/TPRVTGGGAM epitope, was mixed into donor BC262 (HLA-B*0702 negative). Starting at 100% of the BC260 donor, a titration series was generated with fivefold dilutions going from 1.7 to 0.0001% of single, live lymphocytes. Cells were stained with PE- and APC-labeled pMHC multimers and an antibody mix containing a live/dead stain (NIR—Invitrogen), CD8 (PerCP—Life Technologies), and FITC-conjugated dump channel antibodies (CD4, CD14, CD16, CD19, and CD40—BD Biosciences) in order to identify CD8^+^MHC multimer^+^ T cells (2). For spike-in 2, one PBMC sample from donor B1054 (HLA-A*0201 positive) was mixed into donor B1060 (HLA-A*02 negative) in nine steps using twofold dilutions. Sample 1 contained only cells from B1054 with high and intermediate frequencies of T cells responsive toward the CMV HLA-A*0201/NLVPMVATV and FLU HLA-A*0201/GILGFVFTL epitopes, respectively. Sample 9 contained only cells from B1060. Cells were stained with PE-labeled CMV multimer and APC-labeled FLU MHC multimer.

### MHC Multimer Proficiency Panel

FCS files used in this study were from 28 different laboratories who participated in an MHC multimer proficiency panel organized by Immudex. Originally, 51 labs participated in the proficiency panel but only 28 labs made their FCS files available for our analysis. The individual labs were anonymized and given an ID number. Each lab received two PBMC samples from each of two donors—518 and 519—and MHC Dextramers specific for EBV HLA-A*0201/GLCTLVAML, FLU HLA-A*0201/GILGFVFTL or an irrelevant peptide HLA-A*0201/ALIAPVHAV (NEG). Each lab used their own antibodies, staining protocols, and gating strategies, which varied significantly from lab to lab. As a result, the number and type of parameters included by each lab varies to a great extent, but as a minimum all labs included CD3, CD8, and multimer staining or dump, CD8 and multimer staining, using various antibodies. The two donors used held T cell responses against the EBV and FLU-derived T cell epitopes, including both low-frequency responses (0.04 and 0.09% multimer^+^ CD8^+^ T cells), a medium (1.13% multimer^+^ CD8^+^ T cells), and a high-frequency response (5.33% multimer^+^ CD8^+^ T cells) as defined by a pretest on eight donor samples performed at two different locations with insignificant variation. All samples were run in duplicates giving a total of 12 FCS files from each lab. All labs gated their files manually and reported the percentage of identified multimer^+^ CD8^+^ T cells of the total number of CD8^+^ cells. The percentage of MHC multimer^+^ T cells was reported as the mean of the duplicate analysis. Exceptions to this were lab 104 which only provided files from one analysis run, as well as lab 235 and lab 240 where the 518-EBV and 519 FLU samples, respectively, were only included in one run. For these labs, the value from the single run was used instead of the mean value.

### Central Manual Gating

A central manual gating was performed on all FCS files by one operator. SSC-A/FSC-A was used to identify lymphocytes and FSC-H/FSC-A to identify singlets. Of the 28 labs in this study, 17 labs included a live/dead stain in their analysis and 11 did not. From single, live lymphocytes or single lymphocytes the number of CD3^+^, CD8^+^, and MHC multimer^+^ cells were identified and reported. The percentage of multimer^+^ T cells was calculated both from CD8^+^ cells and from total single (live) lymphocytes. For lab 215, the live/dead stain was included in a dump channel stain (CD14, CD16, and CD20); thus, the percentage of multimer^+^ T cells was calculated from single, live, non-dump lymphocytes. The percentage of multimer^+^ T cells reported was the mean percentage calculated from the duplicate analysis. FACS DIVA 8.0 software (BD Biosciences) was used for manual gating and the gated FCS files were exported in FCS 2.0 format.

### Manual Pregating

Prior to automated analysis in FLOCK and SWIFT, the FCS files were gated manually in order to select single lymphocytes or single live lymphocytes (when a live/dead stain was included). Throughout the study, the term pregating is used when referring to manual pregating.

### Manual Postgating

SWIFT analysis was performed on raw FCS files and cluster gating was performed on the SWIFT output files to obtain single lymphocytes or single live lymphocytes (when a live/dead stain was included) before identifying the multimer population as described in the SWIFT pipeline section. Throughout the study, postgating is used when referring to manual postgating.

### Automated Prefiltering

Automated prefiltering was included as an automated alternative to manual pre- or postgating. The same selection was applied as described for manual pregating. The automated prefiltering method we developed for FLOCK and SWIFT, named Directed Automated Gating (DAG), is a 2D by 2D density-based data prefiltering method. The sequence of the 2D dot plots used in the DAG prefiltering is specified in a user-configurable file, which also includes coordinates of a rectangle gate on the 2D dot plot. DAG automatically calculates a set of density contour lines based on the data distribution on the 2D dot plot. The events that are inside the largest density contour line within the rectangle gate will be kept and passed to the next filtering step, until the sequence of the 2D dot plots is fully traversed. DAG is implemented in Matlab and is publicly accessible at Github under GPL3.0 open source license.^3^ Throughout the study, the term prefiltering is used when referring to automated prefiltering.

### FLOCK Pipeline

FCS files were uploaded to FLOCK at www.immport.niaid.nih.gov and joined in datasets for each individual lab. The files were then initially analyzed as a dataset using FLOCK version 1.0 with the parameters set at auto. Unused markers/channels were excluded from the FLOCK analysis as were scatter parameters and parameters that were part of the manual or automated prefiltering. All other parameters included in the stainings performed by individual labs, which were as a minimum CD3, CD8, and MHC multimer or dump, CD8, and MHC multimer, were used for clustering. FLOCK then automatically assigned the values 1–4 (1: negative, 2: low, 3: positive, 4: high) for categorizing expression levels of each marker based on the relative expression level of the given marker on each identified cell population. A file with a large and easily definable MHC multimer^+^ population (in most cases the 519 EBV sample) was then chosen to be a reference sample and the centroid information for this sample was saved. Using the cross-comparison feature, the other samples were then analyzed again with the centroid from sample 519 EBV as a reference. From the output of cross comparison, the summary table was downloaded and imported into excel where the intensity level of each marker in each population was used to define the MHC multimer^+^ population. In order to identify which FLOCK clusters are the CD8^+^, MHC multimer^+^ cells, the expression level cutoff was set at >1 for CD3 (not included in all labs), >1 for CD8, and >2 for MHC multimer. The percentage of MHC multimer^+^ cells of the total single, live lymphocyte population was then calculated and noted, and the mean percentage calculated from the duplicate analysis. The same cutoff value could not be used to identify the CD8 population in samples coming from different labs most likely due to the large variation in fluorochromes used to stain for CD8 cells between individual labs. The cutoff value for the CD8 marker was consequently set very low (>1), including also cells with low CD8 expression into the CD8 population. In many samples, this lead to the inclusion of too many cells into the CD8 population, thereby skewing the frequency of MHC multimer^+^ cells when calculated as a percentage of the CD8 population. As a consequence, the CD8 marker was used only for identifying the true MHC multimer-binding population and not as the base for calculating the frequency of the population, which was instead done using the number of live, single lymphocytes. All FCS files from the 28 labs were analyzed using FLOCK. For three labs (105, 215, and 253), FLOCK analysis resulted in the identification of MHC multimer populations in the negative control samples comprising 20–50% of live, single lymphocytes, and the three labs were therefore considered to be extreme outliers and consequently removed from the analysis of the negative samples.

### SWIFT Pipeline

SWIFT version 3 was downloaded through www.ece.rochester.edu/projects/siplab/Software/SWIFT.html and the SWIFT folder was placed in the Matlab folder. In Matlab, the code swift_fcs_combine was used to generate a consensus file of all samples within each lab. In the FCS combine window, 250.000 cells from each of the 12 samples were chosen to be in the concatenated sample, giving a total of 3 × 10^6^ cells. According to SWIFT online tutorials, the optimal range of cell numbers in a sample is 2–5 × 10^6^. For labs where the nomenclature was not consistent between samples within the given lab, the code swift_modify_channels was used to uniformly name the channels in all files, prior to creation of the consensus FCS file. The concatenated consensus file was clustered using the code swift_main, generating a template file that was then used as a reference to cluster all 12 samples from a given lab with the code swift_assign_main. All parameters contained within a given sample were used for clustering, including the parameters that were part of manual or automated prefiltering. The input cluster number was kept at default settings—100 for all labs—and all unused channels/markers or channels included in the prefiltering were unchecked in both the Dims to Cluster and Output Medians columns. The ArcSinh Factors and Percent Noise were kept at default settings for all fluorescence channels. In the end, the output clustered FCS files were analyzed manually using FlowJo version 10 (Tree star) to obtain the number of CD3, CD8, and MHC multimer^+^ cells or the number of non-dump, CD8, and MHC multimer^+^ cells. Twenty-seven labs were analyzed with SWIFT, lab 208 was left out due to incompatibility of the FCS format with the software. In the analysis of sample 519 FLU for Figure 4C, lab 133 was left out, as it was an extreme outlier.

### ReFlow Pipeline

All FCS files were uploaded on ReFlow and each lab was analyzed individually. The clustering variables assigned were values as follows for both Stage 1 and Stage 2; burn in: 10,000, cluster count: 32, iteration count: 1,000, and sub-sampling count: 20,000. Stage 1 clustering was performed using FSC-A, SSC-A, and live/dead marker (when available). Live lymphocyte clusters were selected manually and Stage 2 clustering was performed using the CD8 and multimer-PE parameters. Singlets were not discriminated in the ReFlow stage 1 clustering as it is not advisable to use more scatter parameters than already used to identify lymphocytes. The multimer^+^ populations were chosen manually based on visual inspection of a 2D (CD8 versus multimer) representation of the clustered data. Frequency of multimer^+^ clusters (sum of frequencies when more than one cluster) were exported as a .csv file and were used for analysis. Out of the 28 labs included in the study, ReFlow was unable to analyze labs 133, 208, 239, and 254 due to compensation issues, thus 24 labs were analyzed with ReFlow. After ReFlow clustering Lab 224 was found to be an extreme outlier and was consequently removed from the statistical analysis, giving a total of 23 labs in the final analysis.

### Analysis and Statistics

The gating analysis that was performed in this study was carried out by two different immunologists. Central manual gating, FLOCK, and SWIFT analyses were performed by NWP whereas ReFlow analysis was performed by AC.

Statistical analyses were performed using GraphPad Prism 7 and R 3.3.2. A paired *t*-test was used to test for differences among the different algorithms, and correlations were calculated using Pearson correlations. In R, the package cvequality_0.1.1 was used to perform an asymptotic coefficient of variation (CV) equality test. For all tests, it was assumed that the data were sampled from Gaussian populations. The normal distribution was explored in R using a boxcox transformation, suggesting a log transformation of the data. All statistical tests were therefore also performed on log transformed data but gave the same results, except for the asymptotic CV test in Figure 4B. When using the log transformed data, FLOCK and ReFlow software also resulted in significantly higher variation compared with manual gating for the 519 FLU population.

## Results

### Individual Gating as a Source of Variation in the Assessment of MHC Multimer-Binding T Cells

**Figure 1:**
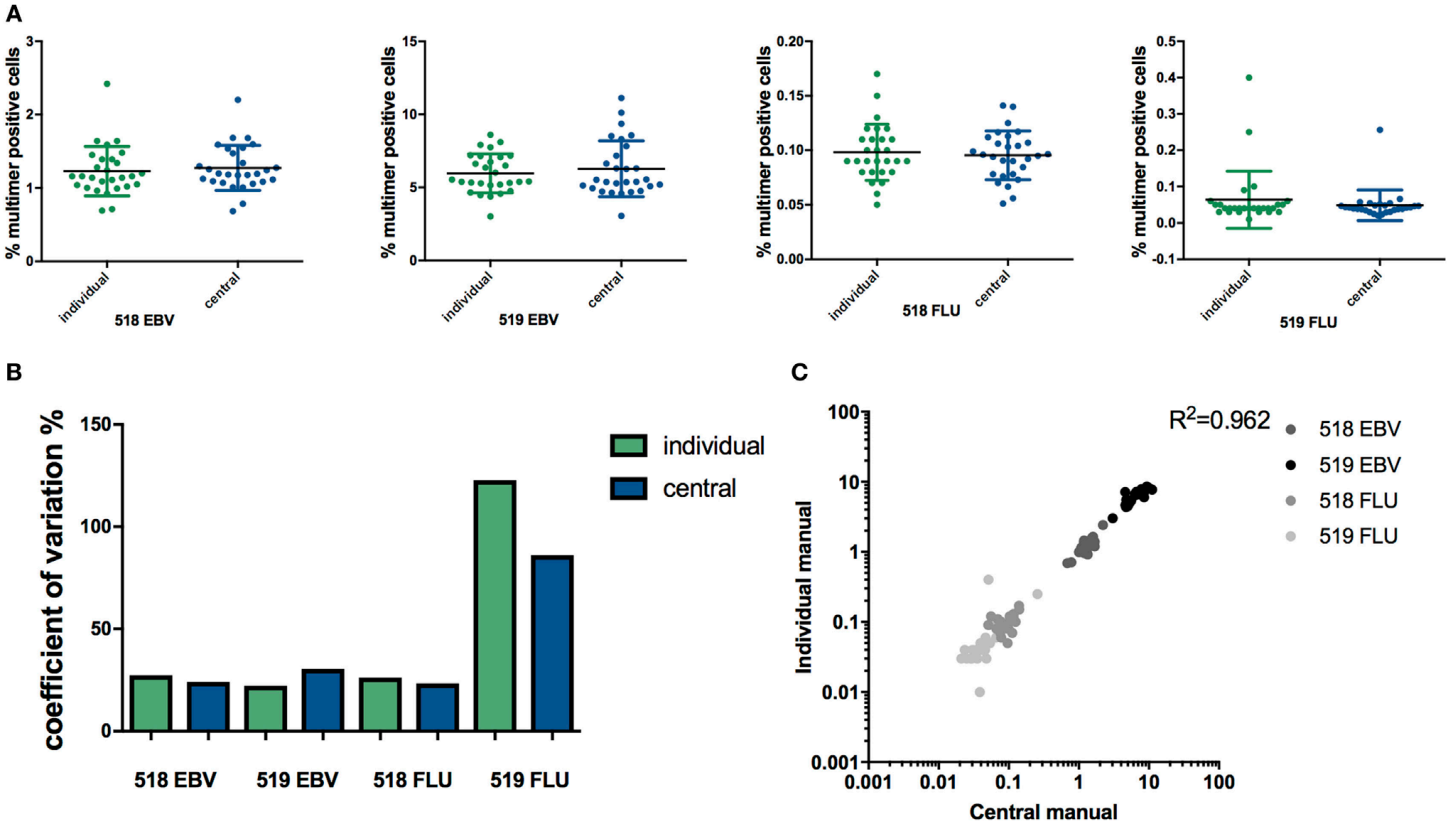
Individual versus central manual gating. **(A)** Percentage of multimer positive cells (EBV or FLU) in total CD8^+^ T cells in two healthy donors (518 and 519) identified through individual or central manual gating. Each dot represents the mean value for duplicate experiments for an individual lab, *n* = 28. Line indicates mean and error bars indicate SD. No significant difference between individual gating and central gating was detected (paired *t*-test). **(B)** The coefficient of variation (CV = SD/mean*100) related to the identification of major histocompatibility complex multimer positive T cell populations either through individual gating (green) or central manual gating (blue) for the two virus responses and two donors. No differences are statistically significant (asymptotic CV equality test). **(C)** Correlation of the percentage of multimer positive cells found with individual and manual gating. *p* < 0.0001 (Pearson correlation), *n* = 112. Mean values from duplicate experiments are shown. Different colors represent different populations. Individual: gating is done by each individual lab. Central: gating on all files is performed by the same person. 519: healthy donor 519; 518: healthy donor 518; EBV: Epstein–Barr virus; FLU: influenza virus.

To assess the impact of individual manual gating compared with central manual gating on specific T cell identification and quantification, FCS data files obtained from the MHC multimer proficiency panel were re-analyzed manually by the same operator. The frequency of MHC multimer^+^ cells within CD8^+^ cells, reported by each lab (individual manual analysis) was compared with the respective frequencies determined after central manual analysis. For all four cell populations: 518/EBV, 519/EBV, 518/FLU, and 519/FLU, no significant difference in the determined frequency was observed between manual individual and central gating (Figure 1A). The highest CV was observed for the lowest frequency (519/FLU) population, but no statistically significant difference between individual and central manual gating was found (CV = 122% and CV = 86%, respectively) (Figure 1B). Previous data have shown that centralizing the gating may reduce the %CV compared with individual gating (9). Furthermore, a recent publication reported a similar observation that the infrequent and poorly resolved cell populations can be highly variable across samples when individual manual gating analysis is used (21). Additionally, our results show a linear correlation between central and individual gating throughout the range of T cell frequencies analyzed (Figure 1C). Throughout the remaining study, the values from central manual analysis were used when comparing automated and manual flow cytometry analyses.

### Performance of Automated Software

We next evaluated the ability of the three automated gating algorithms FLOCK, SWIFT, and ReFlow to identify MHC multimer-binding T cells. Each algorithm varied with respect to the processing time, additional software requirement, manual handling before or after the automated processes, and annotation requirements. Relevant features of the selected algorithms have been listed in Table 1. Specifically, substantial manual handling may impact both the objectivity and handling time—two parameters that we aim to improve through computational analysis. The workflow for each automated analysis tool is depicted in Figure S1 in Supplementary Material.

**Table 1:** Features of the three software solutions.

Feature	SWIFT	FLOCK	ReFlow
Availability	Free but requires Matlab	Free online	Free online
Program run time	~1 h	~10 min	~30 min
Template feature	Yes	No	Yes
Cross-comparison feature	Yes	Yes	Yes
Difficulties in output analysis	New gating method—centroid cluster gating	Choosing cutoff values	Easy
Automatization	+	+++	++
Sensitivity	+++	+	++
Requires common nomenclature of parameters	Yes, renaming of channels is possible	Yes	Yes, harmonized by the tool
Repository	No	No	Yes
Hardware requirement	Runs locally on the computer—analysis speed depends on local computer resources	Web access—analysis speed depends on FLOCK compute resources	Web access—analysis speed depends on ReFlow compute resources
Feasibility for non-computational experts	+	++	+++

*Program run times represent the time it takes the software to analyze all files within one lab. For Scalable Weighted Iterative Flow-clustering Technique (SWIFT), it includes the clustering of a consensus sample and subsequent clustering of all samples based on the template*.

**Figure 2:**
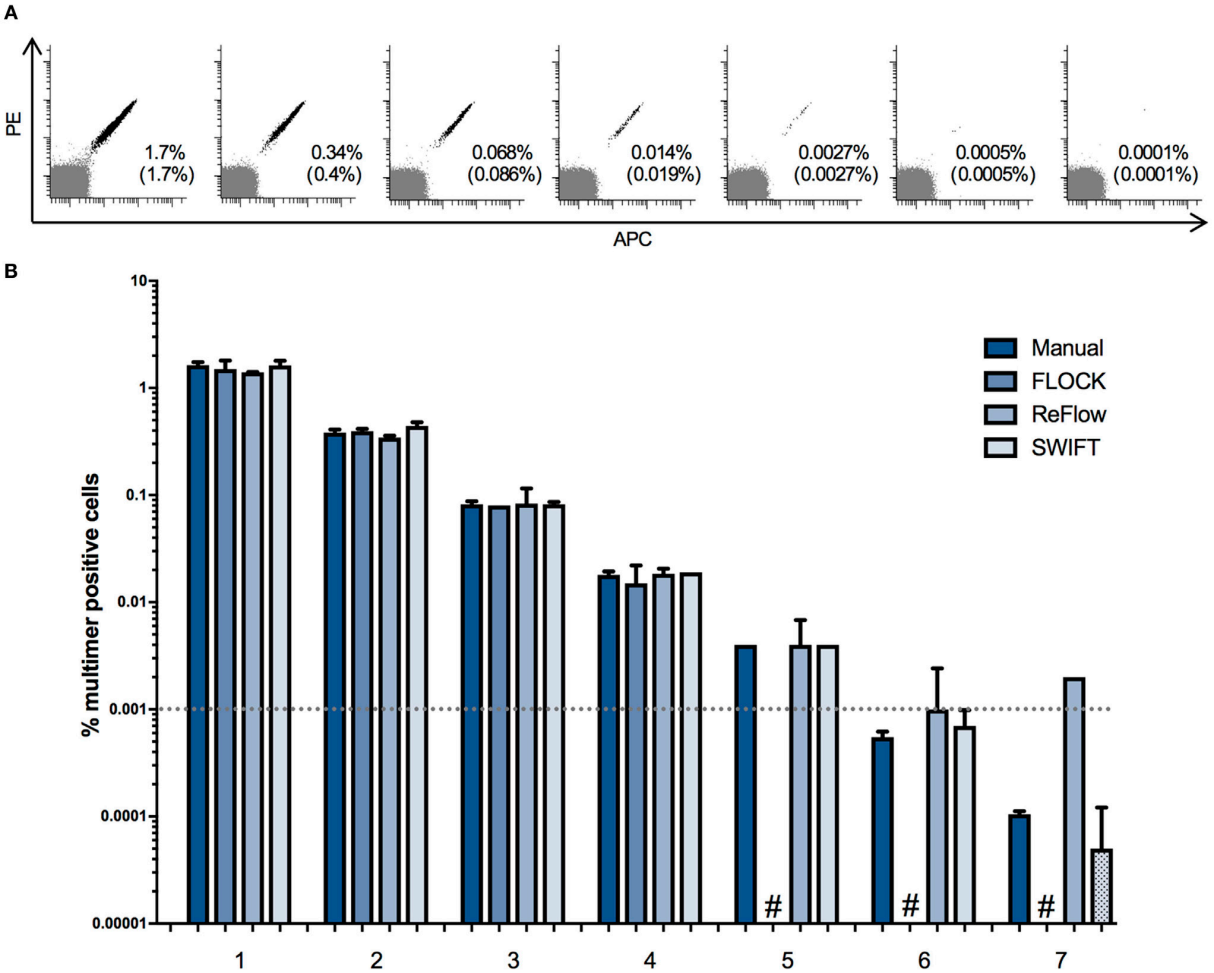
Limit of detection for different automated approaches. A donor carrying ~1.7% CD8^+^ T cells binding to HLA-B*0702 cytomegalovirus (TRP) was spiked into an HLA-B0702 negative donor in fivefold dilutions in order to assess the limit of detection of the four analysis approaches. The experiment was run in duplicates. **(A)** Dot plots of the spiked samples showing the theoretical frequency of multimer + cells of the total lymphocyte population and the actual detected frequency (in brackets) by manual gating. Multimer + cells are double positive for PE and APC. PE: phycoerythrin; APC: allophycocyanin. **(B)** The mean percentage of multimer positive cells out of single, live lymphocytes. Numbers represent the seven different samples. Dotted bars: the software detected zero specific cells in one of the two duplicates. #: the software was unable to detect the specific populations in both duplicates. Dashed line: a typical detection threshold for positive response in a major histocompatibility complex multimer staining.

First, we addressed the limit of detection for the three selected algorithms, through analysis of two independent titration experiments. We used PBMCs from one donor (BC260) carrying 1.7% HLA-B0702 CMV_TPR_-specific T cells in total live lymphocytes and mixed this in fivefold dilution steps with an HLA-B702 negative donor (BC262). A total of seven serial dilutions were used, giving a theoretical frequency of MHC multimer^+^ cells ranging from 1.7 to 0.0001% out of total live, single lymphocytes, and each sample was analyzed by flow cytometry for the presence of HLA-B*0702 CMV_TPR_ multimer-binding CD8^+^ T cells (Figure 2A). Secondly, a titration curve was generated by mixing a PBMC sample from donor B1054 holding an HLA-A*0201 CMV_NLV_ and an HLA-A*0201 FLU_GIL_ response of 0.87 and 0.13% of total lymphocytes in twofold dilution steps with donor B1060 (HLA-A*0201 negative). A “negative sample” of PBMCs from B1060 alone was also included (Figure S2 in Supplementary Material). The FCS files were analyzed, using manual analysis, FLOCK, SWIFT, and ReFlow software tools. Frequencies of MHC multimer^+^ cells were not compared based on CD8^+^ cells because there was no consistent CD8 expression cutoff value to use in annotating the data clusters identified by FLOCK. The same cutoff value could not be used across samples coming from different labs most likely due to the large variation in antibodies/fluorochromes used to stain for CD8 cells between individual labs. Hence, to enable comparison of results between all analysis methods, the frequency of MHC multimer-binding T cells was calculated based on live, single lymphocytes.

Our data show that all three algorithms perform equally well in comparison with central manual gating in identifying populations >0.01% of total lymphocytes (Figure 2B; Figure S2 in Supplementary Material). At frequencies <0.01%, FLOCK either assigned too many cells to the MHC multimer population or did not associate any cell population with MHC multimer binding (Figure 2B; Figure S2 in Supplementary Material). ReFlow also assigned too many cells to the MHC multimer^+^ cluster for the low-frequency populations, resulting in the assignment of approximately 0.002% MHC multimer^+^ cells regardless of their true presence, as these were also assigned in the negative or very low-frequency samples (Figure 2B; Figure S2 in Supplementary Material). Only the SWIFT algorithm was able to identify cell populations of similar sizes as theoretically present and detected through manual analysis, down to the range of 0.0005–0.0001% of total lymphocytes, where only one to five events were present on the corresponding dot plots (Figure 2A). For manual analysis, a threshold of 10 events is usually applied, corresponding to 0.001% of total lymphocytes in these samples (represented by the dashed line in Figure 2B). However, for high avidity T cells that are very well separated based on fluorescence intensity, as in this case, the presence of MHC positive T cells can be followed at even lower frequencies.

### Automated Analysis of MHC Multimer-Binding T Cells from Proficiency Panel Data

In order to reduce noise from irrelevant cell populations a preselection of live, single cell lymphocytes was performed prior to the automated analysis. We compared manual pregating to an automated prefiltering process using DAG (see footnote text 3), for its impact on the following identification of MHC multimer^+^ T cells using either FLOCK or SWIFT. The final assessment of MHC multimer^+^ T cells was not affected by the choice of pregating strategy, and the obtained data correlated tightly throughout the range of MHC multimer^+^ T cell frequencies analyzed (Figure S3 in Supplementary Material). Since ReFlow includes a separate build-in prefiltering process, the impact of the preselection methods was consequently not compared.

**Figure 3:**
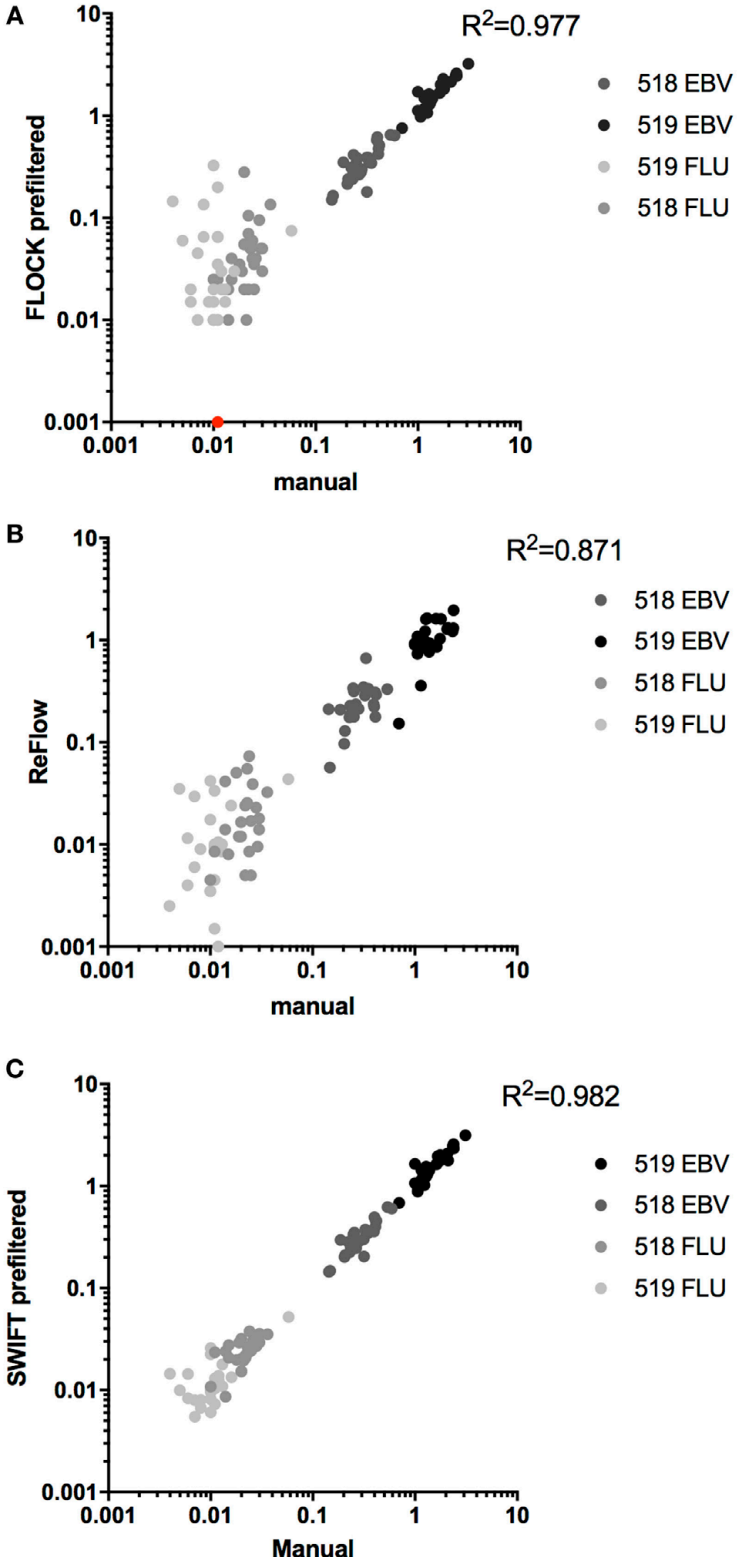
Automated analyses versus central manual gating. Correlation between automated analyses and central manual gating for the identification of MHC multimer positive T cell populations, using either of the three algorithms: **(A)** FLOCK, *n* = 112, *p* < 0.0001, one data point of 0% was converted to fit the log axis (given in red); **(B)** ReFlow, *n* = 92, *p* < 0.0001; **(C)** SWIFT, *n* = 108, *p* < 0.0001. All *p*-values are Pearson’s correlations. Different colors indicate different populations.

Next, we compared the identification of MHC multimer-binding T cells across the three automated analysis tools to central manual analysis of the proficiency panel data. The number of relevant MHC-binding T cells was assessed for both donors: donor 518, EBV (~0.3%), FLU (~0.02%), and donor 519 EBV (~1.5%), FLU (~0.01%), all values are given as %MHC multimer-binding T cells out of total live, single lymphocytes. The coefficients of determination (*R*^2^) for the three correlations were calculated separately for the high-frequency populations (518 and 519 EBV), for the low-frequency responses (518 and 519 FLU), and for all populations together. Overall, the three algorithms were able to identify most of the MHC multimer-binding T cell populations in a similar range as identified by manual gating (FLOCK: *R*^2^ = 0.977, ReFlow: *R*^2^ = 0.871, SWIFT: *R*^2^ = 0.982) (Figures 3A–C). However, a spreading was observed for low-frequent T cell populations, especially using FLOCK and ReFlow (Figures 3A,B). For FLOCK, the correlation was tight for the high-frequency populations (*R*^2^ = 0.965) but a significant spreading was observed for low-frequency populations (*R*^2^ = 0.00676) (Figure 3A). There were two different issues giving rise to this observation: one was that for the low-frequency populations, FLOCK assigned background events into the true MHC multimer^+^ T cell population. The other issue was related to the difficulty of annotating the data clusters identified in the FLOCK analysis. As a fully automated unsupervised clustering method, FLOCK assigned the values 1–4 (1: negative, 2: low, 3: positive, 4: high) for categorizing expression levels of each marker based on the relative expression level of the given marker on each identified cell population. In this study, an MHC multimer^+^ T cell population was defined as having an expression level >1 for CD3 (not included in all labs), >1 for CD8, and >2 for the MHC multimer. The same cutoff value was used for all samples in order to have a standardized analysis pipeline, requiring a minimum of manual intervention. The chosen cutoff value was however not suitable for all samples, as there were cases where populations that by visual inspection were defined as clearly MHC multimer^-^, were identified by FLOCK as multimer^+^ populations based on the cutoff values applied. These populations resulted in a false positive assignment of MHC multimer^+^ T cells. This was particularly the case for samples holding low-frequency MHC multimer^+^ T cell populations (Figure S3 in Supplementary Material). ReFlow showed a larger spreading throughout the range of T cell frequencies but—like FLOCK—had better performance when detecting high-frequency populations (*R*^2^ = 0.776) as opposed to low-frequency populations (*R*^2^ = 0.138) (Figure 3B). For SWIFT analysis, a tight correlation was observed for both high-frequency and low-frequency populations (*R*^2^ = 0.968 and 0.722, respectively) (Figure 3C).

**Figure 4:**
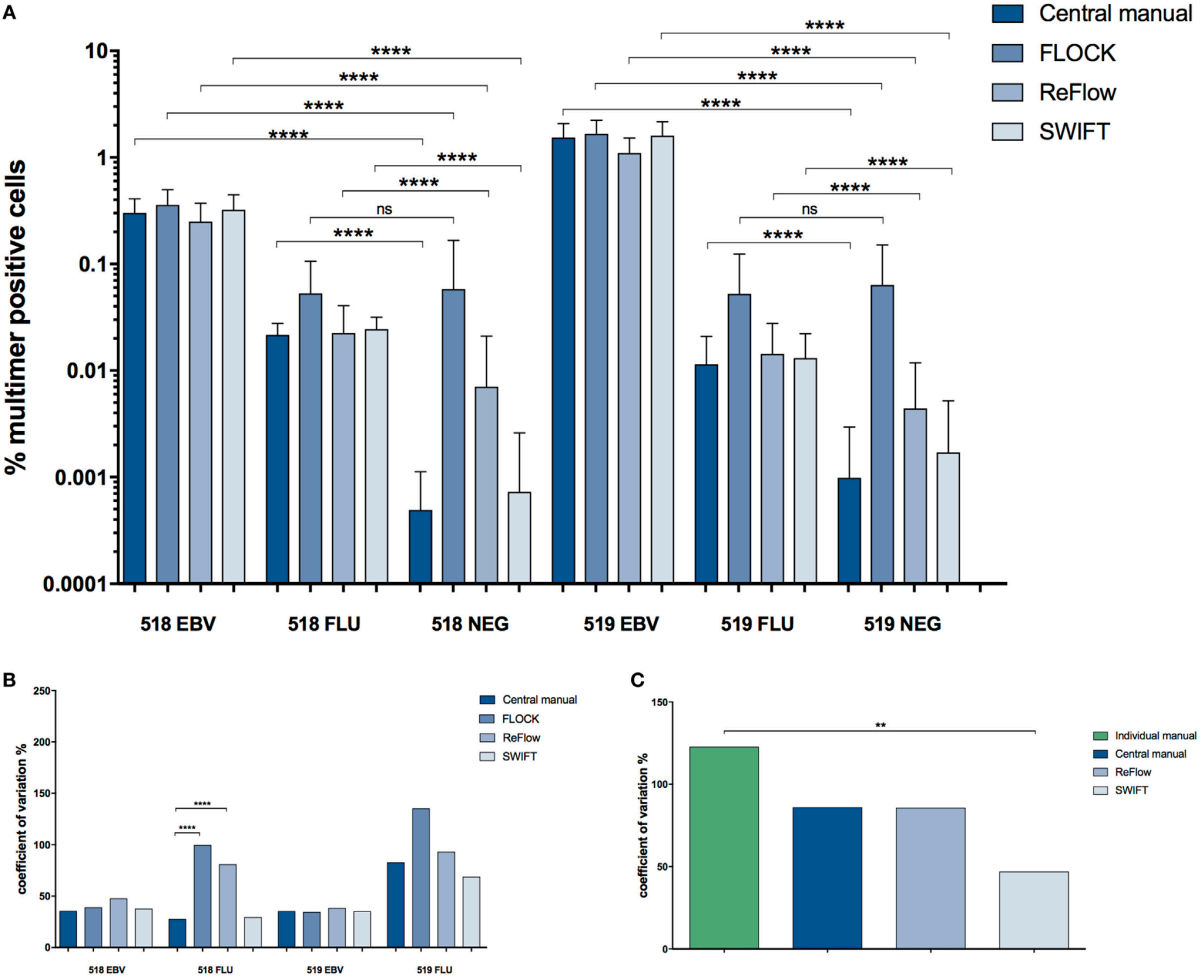
Comparison of the different analysis methods. **(A)** Percentage of MHC multimer^+^ T cells out of single, live lymphocytes found using the different analysis approaches for identification of T cells recognizing two different virus-derived epitopes (EBV, FLU) in two different donors (518, 519). Error bars indicate SD. ****: *p* < 0.0001; ns: not significant (paired *t*-test). Central manual: *n* = 28, FLOCK: *n* = 28, ReFlow: *n* = 23, SWIFT: *n* = 27. **(B)** The coefficient of variation (CV) (SD/mean*100) for the different analysis approaches in determining the frequency of MHC multimer^+^ T cells. ****: *p* < 0.0001; no line: no significant difference (asymptotic CV equality test). **(C)** The CV (SD/mean*100) specifically related to the FLU-specific response in donor 519. **: *p* < 0.01; no line: no significant difference (asymptotic CV equality test). For **(C)**, the CV is calculated based on percentage of MHC multimer^+^ T cells out of total CD8 T cells in order to compare with individual manual gating. 518: healthy donor 518; 519: healthy donor 519; EBV: Epstein–Barr virus; FLU: influenza virus.

In order to compare the automated analysis tools to each other, we determined the average frequency of the different MHC multimer-binding T cell populations identified and the CV obtained when using either central manual gating, FLOCK, SWIFT, or ReFlow (Figures 4A,B). Again, all evaluated tools could identify high and intermediate frequency T cell populations (518/EBV and 519/EBV) with low variance and significantly differentiate these from the negative control sample (Figure 4A). The low-frequency populations (518/FLU and 519/FLU) could, however, not be distinguished from the negative control samples by FLOCK. For ReFlow, a significant difference between the EBV- or FLU-specific T cell holding samples and the negative control sample was obtained; however, the assigned number of MHC multimer-binding cells in the negative samples was higher compared with both central manual analysis and SWIFT analysis (Figure 4A). SWIFT analysis enabled identification of the low-frequency MHC multimer-binding T cell populations at equal levels to the central manual gating (Figure 4A). In terms of variance, similarly, SWIFT provided comparable variance in the determination of low-frequency MHC multimer-binding T cells (FLU in 518 and 519), compared with central manual gating. In contrast FLOCK, and to a lesser extend ReFlow, resulted in increased variation for the low-frequent responses which was statistically significant only for the 518 FLU response (Figure 4B).

We finally assessed if the use of automated analyses could reduce the variation in identification of MHC multimer^+^ T cell populations when compared with the individual manual gating conducted by the different labs involved. We chose to look at the smallest population in our study, the donor 519 FLU population as this population had the highest variance. In order to make this assessment, we needed to assign the frequency of the MHC multimer^+^ population based on the CD8^+^ T cells. Consequently, this was evaluated exclusively for ReFlow and SWIFT, as the assignment of the correct CD8^+^ population was challenging on this dataset using the FLOCK algorithm based on the uniform criteria’s that were chosen across the full data set and the high inter-lab variations (see Materials and Methods). The variance was assessed by comparing the CV for the frequencies found with individual manual gating, central manual gating, and the two automated analysis tools (Figure 4C). This comparison showed that automated gating analysis using SWIFT provided significantly lower variance compared with individual gating, which is the situation applied to most data analyses. ReFlow analysis lowered the variance to the same level as central manual gating, although this was not statistically significant.

## Discussion

In this study, we evaluated the feasibility of using automated gating strategies for the detection of antigen-specific T cells using MHC multimers. Among the three algorithms tested, FLOCK, SWIFT, and ReFlow, all proved useful for automated identification of MHC multimer^+^ T cell populations from the proficiency panel at levels >0.1% which was also reflected in the high degree of correlation of all the tools with central manual analysis. Detection of responses with frequencies in the range of 0.05–0.02% within living lymphocytes was also feasible with SWIFT and ReFlow; however, only SWIFT algorithm was able to detect cell populations <0.02%. The detection limit of ReFlow was lower based on the spike-in experiments (0.002%) and one possible explanation for this discrepancy is the difference in the intensity of the pMHC positive population and the quality of the cell samples. The samples acquired during the spike-in experiment showed a very distinct MHC multimer population and almost no background, whereas the samples acquired for the proficiency panel showed a larger variation in terms of background and fluorescent separation of the MHC multimer population. This finding highlights the importance of sample quality and fluorescent separation when using automated analysis tools. The lower limit of detection of SWIFT is consistent with the results of the FlowCAP II challenge where SWIFT was one of the top performers in the identification of rare cell populations (12). However, in a more recent study that compared automated analysis tools in a fully automated fashion (i.e., no cluster centroid gating allowed), SWIFT was outperformed by other algorithms that were not tested in this study (13). In this particular study, all tested algorithms were compared in a fully automated fashion, which is not the way SWIFT was applied in our study. Here, SWIFT clustered output files were further gated manually on cluster centroids. This might explain the discrepancy between these and our results, and also suggests that centroid gating may improve analysis of automated clustering results. An alternative to the manual gating step could be to run the SWIFT clustered output files in another algorithm, which could potentially also improve the automated analysis as was seen in the FlowCAP I challenge where the best results were obtained when the algorithms were combined (12). The dataset analyzed here, holds a large diversity in terms of antibodies and fluorescent molecules used for the identification of CD8^+^ T cells. As such this dataset represents a “worst case scenario” for automated gating algorithms. Consequently, it was impossible to normalize staining intensities to a given standard, and cross-sample comparison could only be applied within each lab. This lack of standardization may impact the performance of the different algorithms. However, the ability to work across large differences in assay design is necessary to compare flow cytometry data between various laboratories. Obviously, when multicenter immunomonitoring projects are planned, it is advantageous to harmonize staining protocols and antibody panels across different laboratories, and such harmonization will ease the following automatic analyses and improve the outcome.

In terms of handling the three software tools, a number of relevant differences should be highlighted. FLOCK has a very user-friendly web interface with several different analysis features. The output is graphically very similar to regular dot plots and as such is well recognized by immunologists and easy to interpret by non-computational experts. An additional strength of FLOCK is the possibility to manually adjust the centroids chosen by the algorithm, in cases where they were obviously misplaced. In this study, we did not interfere with the FLOCK analysis as we aimed to obtain a standardized and fully automated approach. The ability to make manual adjustments combined with a clear graphical readout provides a sense of transparency and understanding of the analysis process, making it attractive to immunologists with limited computation expertise. Since the completion of this study, the FLOCK platform has been updated to include even more analysis features, further improving the FLOCK interface. Finally, as stated in Table 1, FLOCK analysis is quite fast especially compared with SWIFT. However, prior to FLOCK analysis, FCS files must be uploaded to the web interface, which can be time consuming depending on file size. The SWIFT algorithm runs locally on the computer through Matlab and consequently requires a minimal level of coding abilities. All codes are well described in the manual associated with the SWIFT installation files and simple to use. SWIFT does not require data-upload to a distant server, but may require substantial run times, depending on the local computer power. However, the slower initial clustering of a consensus file is partly compensated by the rapid assignment of individual samples to the initial cluster template. Similar to FLOCK, the SWIFT algorithm allows adjustment of parameters important for the analysis output, like input cluster number, ArcSinh Factors, and Percent Noise. These features are, however, not very intuitive for non-computational experts to understand and hence challenging to adjust in a meaningful manner. The output files generated by SWIFT, when analyzed in, e.g., FlowJo, can be displayed as either conventional dot plots, or as somewhat different dot plots in which each dot represents a full cluster rather than a single cell. This feature provides some flexibility, allowing an operator more freedom to position gates and still catch the target population across samples, even in the presence of machine noise or slight fluorescence shifts. Thus, SWIFT provides a clustering of events, but the final binning of various clusters into certain parameter-defined categories is done through manual cluster gating (in the present study) or can be accomplished by a second automated platform (17). ReFlow also has a simple and intuitive user interface that is accessible via a standard web-browser. It requires no programming knowledge to learn and operate. The FCS files have to be uploaded on to the server at speeds determined by the local internet connection. FCS files that belong together are analyzed as a group and since this is performed on shared GPUs, it is not affected by the local computational hardware. Results can be visualized graphically as 2D dot plots (showing both clusters as well as events within clusters) and in tabular format that can be further exported into a csv file. From the graphical view, clusters of interest may manually be further selected, named, and evaluated or may be selected for a further second stage analysis, as it was performed for the current study. Live, lymphocytes were chosen for a further round of clustering to determine multimer positive clusters that are then chosen based on visual inspection of the clusters. The manual selection of clusters in ReFlow is somewhat easier than cluster gating on SWIFT output data, as it is an incorporated part of the algorithm and can be done directly from the analysis.

None of the three automated gating algorithms tested in this study provide a fully automated pipeline. Whether it is choosing cutoff values in FLOCK, cluster gating in SWIFT or choosing positive populations by visual inspection in ReFlow, the analysis of the clustering output requires some manual decision making. That being said, the manual cluster gating performed on the SWIFT files was more laborious than what was needed for the other algorithms. In this study, the FLOCK pipeline was the most automated process as the same cutoff values were applied to all samples. In fact, it might very well have improved the FLOCK analysis if the cutoff level had been defined for each individual sample—which would have been similar to the process for SWIFT and ReFlow. With such sample-specific adjustments, at least one of the issues depicted in Figure S4 in Supplementary Material would have been eliminated. Hence, the FLOCK algorithm provides an analysis platform with higher degree of automatization, but this comes at the expense of sensitivity at least for this very diverse dataset.

A few things are worth considering if a more automated approach is desired, such as harmonization of the staining reagents and procedure, data collection, and FCS file management. In this study, we believe it would have improved the results from the FLOCK analysis had the same antibody been used for the given markers across different labs. This would have eliminated some of the discussed issues with setting an appropriate cutoff level as the fluorescence intensities could have been normalized and would also have allowed the cross-comparison feature to be applied to all samples at once instead of as current within each lab individually. Also, the procedure for SWIFT analysis could potentially have been improved by this, as all labs could have been analyzed using the same template file. Additionally, sample quality is an important issue. Just as it is difficult to manually gate samples with a lot of background due to poor cell sample quality or preparation, it makes the automated detection of specific populations equally, if not even more difficult, as the subjective distinction between background and true events based on visual inspection is removed from the analysis process. Furthermore, common parameter nomenclature between FCS files would lead to less manual intervention, eliminating the step of manual adjustment of parameter names, which is an option within most automated tools. The field of computational analysis of flow cytometry data is rapidly developing, leading to increasingly sophisticated tools that can more accurately detect the exact cell populations of interest. This development is an ongoing process dependent on feedback from actual users and exchange between the fields of software development and immunology.

In this study, we particularly aimed to evaluate automated flow cytometry analysis tools that can be used by experienced flow cytometry users with no programming skills. For all three tested algorithms, there were challenges throughout the study, and it is a problem that non-computational experts have limited possibilities to trouble-shoot data analysis in the computational space. This highlights the need for a closer interaction between the two fields of immunology and bioinformatics/programming and also the need for immunologists to educate themselves within the field of bioinformatics in order to keep up with the development of increasingly complex data analysis in the future (10).

The data presented here shows the feasibility and potential advantage of using automated gating strategies, even across very diverse datasets. The algorithms included here, represent three user-friendly tools for such assessment, but it is by no means an exclusive list. Many computational tools for flow cytometry analyses are currently present, each having their own pros and cons and the choice of algorithm depends on the characteristics of the individual experiments and the desired outcome. Thus, it is crucial to choose carefully when deciding which algorithms to use for each purpose (10, 22).

## Author Contributions

NWP performed gating analysis, made figures, analyzed data, and wrote the manuscript; AC performed gating analysis, discussed data, and revised the manuscript; YQ, JR, AL, and RS performed gating analysis and revised the manuscript; KJ and CH provided data files and revised the manuscript; NVP and MH performed gating analysis; RHS, TM, CC, and SW provided technical guidance and revised the manuscript; CG analyzed and discussed data and revised the manuscript; SH conceived the concept, analyzed and discussed data, and wrote the manuscript.

## Conflict of Interest Statement

The authors declare that the research was conducted in the absence of any commercial or financial relationships that could be construed as a potential conflict of interest.

## Footnotes

^1^www.immudex.com/proficiency-panels.

^2^www.CIMT.eu/CIP.

^3^https://github.com/maxqian/DAG.

## Abbreviations

APC, allophycocyanin; CIP, Immunoguiding Program of the Association for Cancer Immunotherapy; CMV, cytomegalovirus; CV, coefficient of variation; DPGMM, Dirichlet process Gaussian mixture model; EBV, Epstein–Barr virus; FLU, influenza; MHC, major histocompatibility complex; TCR, T cell receptor; PBMCs, peripheral blood mononuclear cells; PE, phycoerythrin; pMHC, peptide MHC.
