# Supplementary material for: Automated Analysis of Flow Cytometry Data to Reduce Inter-Lab Variation in the Detection of Major Histocompatibility Complex Multimer-Binding T Cells
Source: Front Immunol. 2017 Jul 26;8:858. doi: 10.3389/fimmu.2017.00858 (PMC5526901; doi:10.3389/fimmu.2017.00858)
Supplement: Supplementary file 1 [file Presentation_1.PPTX]

## Slide 1
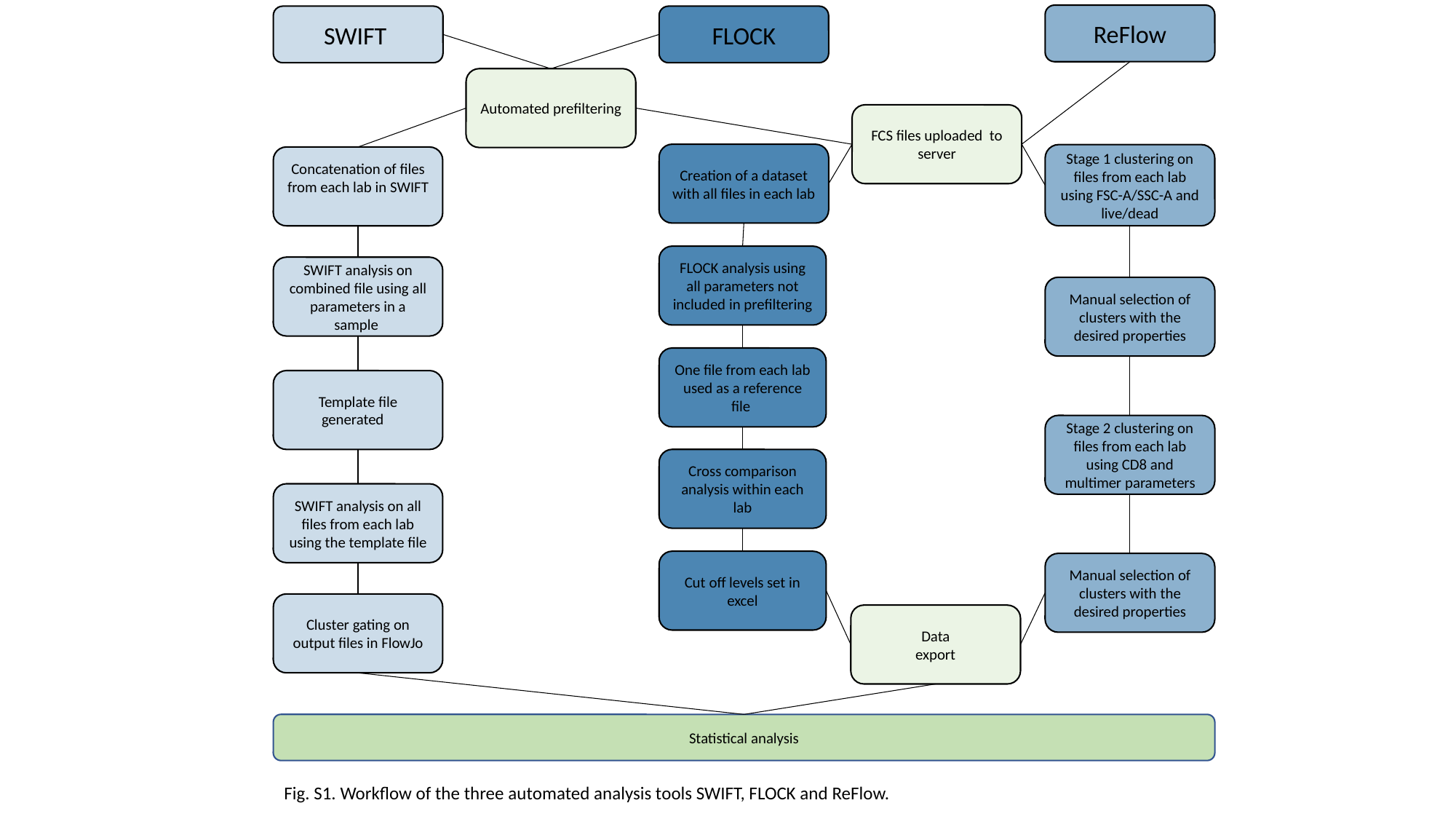

ReFlow
SWIFT
FLOCK
Automated prefiltering
FCS files uploaded to server
Creation of a dataset with all files in each lab
Stage 1 clustering on files from each lab using FSC-A/SSC-A and live/dead
Concatenation of files from each lab in SWIFT
FLOCK analysis using all parameters not included in prefiltering
SWIFT analysis on combined file using all parameters in a sample
Manual selection of clusters with the desired properties
One file from each lab used as a reference file
Template file generated
Stage 2 clustering on files from each lab using CD8 and multimer parameters
Cross comparison analysis within each lab
SWIFT analysis on all files from each lab using the template file
Cut off levels set in excel
Manual selection of clusters with the desired properties
Cluster gating on output files in FlowJo
Data
export
Statistical analysis
Fig. S1. Workflow of the three automated analysis tools SWIFT, FLOCK and ReFlow.

## Slide 2
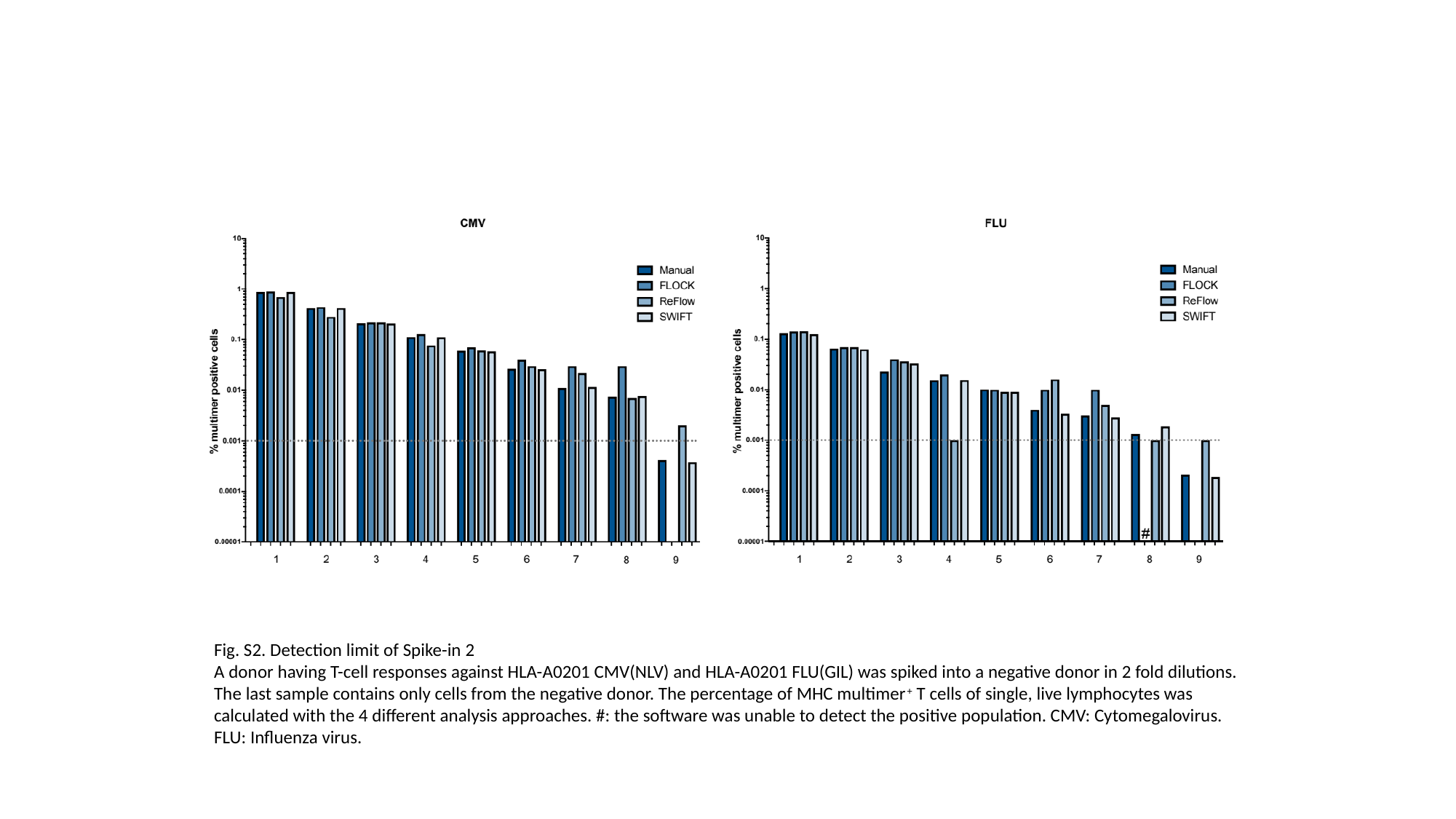

Fig. S2. Detection limit of Spike-in 2
A donor having T-cell responses against HLA-A0201 CMV(NLV) and HLA-A0201 FLU(GIL) was spiked into a negative donor in 2 fold dilutions. The last sample contains only cells from the negative donor. The percentage of MHC multimer+ T cells of single, live lymphocytes was calculated with the 4 different analysis approaches. #: the software was unable to detect the positive population. CMV: Cytomegalovirus. FLU: Influenza virus.

## Slide 3
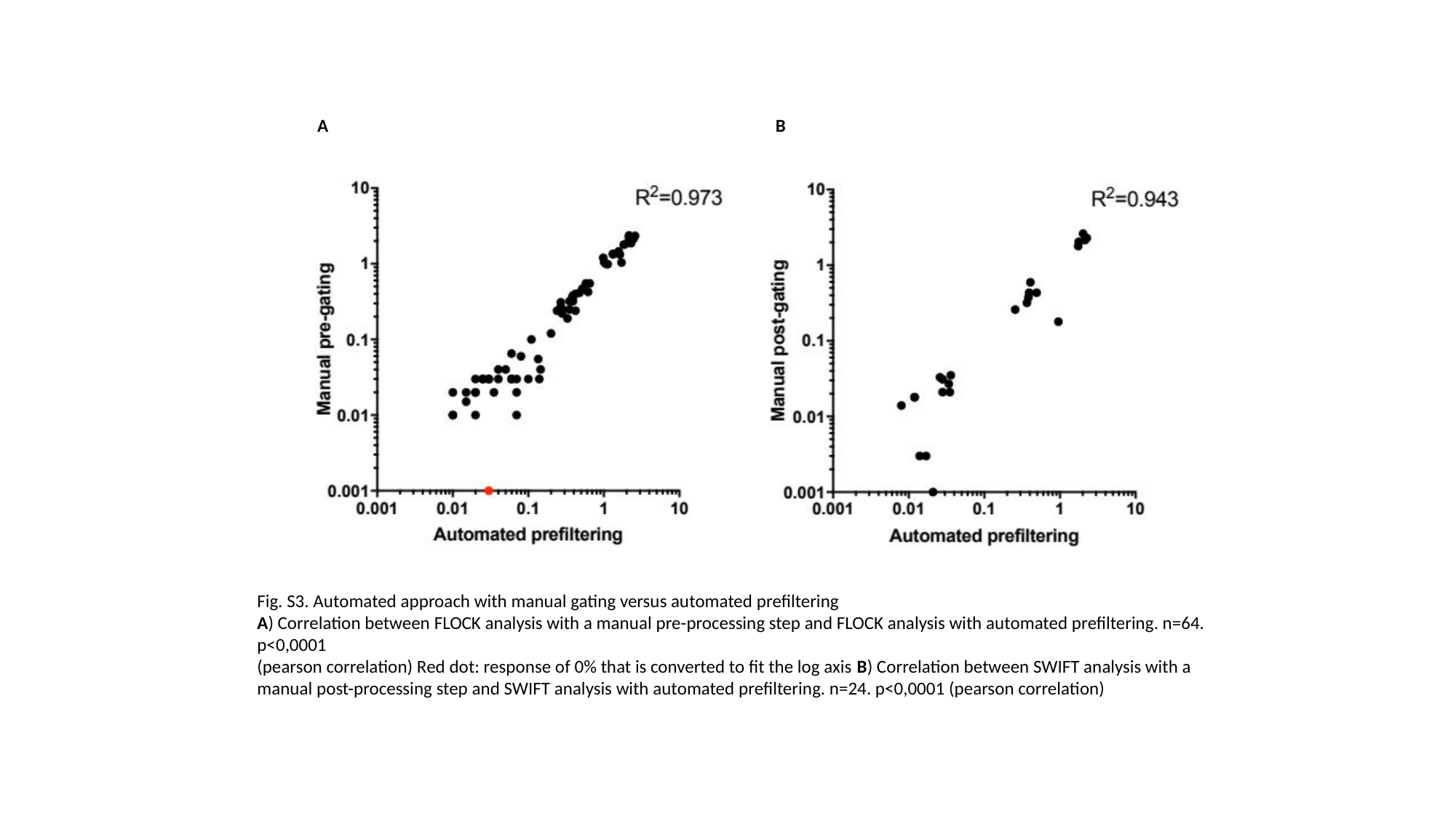

A
B
Fig. S3. Automated approach with manual gating versus automated prefiltering
A) Correlation between FLOCK analysis with a manual pre-processing step and FLOCK analysis with automated prefiltering. n=64. p<0,0001
(pearson correlation) Red dot: response of 0% that is converted to fit the log axis B) Correlation between SWIFT analysis with a manual post-processing step and SWIFT analysis with automated prefiltering. n=24. p<0,0001 (pearson correlation)

## Slide 4
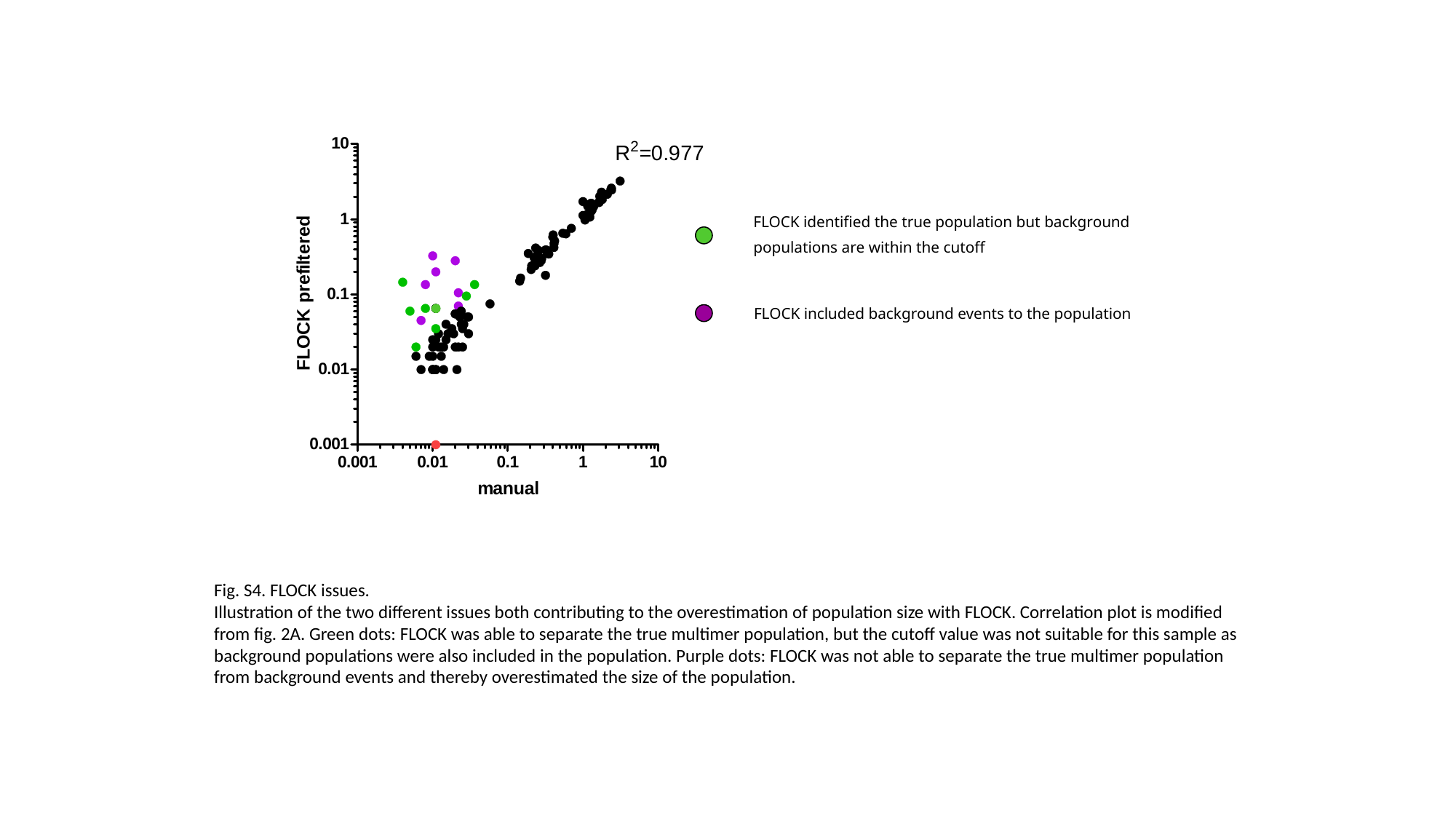

FLOCK identified the true population but background
populations are within the cutoff
FLOCK included background events to the population
Fig. S4. FLOCK issues.
Illustration of the two different issues both contributing to the overestimation of population size with FLOCK. Correlation plot is modified from fig. 2A. Green dots: FLOCK was able to separate the true multimer population, but the cutoff value was not suitable for this sample as background populations were also included in the population. Purple dots: FLOCK was not able to separate the true multimer population from background events and thereby overestimated the size of the population.
